# Group Health & Wellness Coaching: development and validation of the required competencies

**DOI:** 10.1186/s12913-024-10704-x

**Published:** 2024-03-28

**Authors:** Ruth Q Wolever, Timothy R Cline, Jocelyn M Weiss, Suzie Carmack, Cindy Schultz, Michael Arloski, Karen Lawson

**Affiliations:** 1 Department of Physical Medicine & Rehabilitation, Vanderbilt University Schools of Medicine and Nursing, Nashville, TN USA; 2 Dr. Tim Cline, LLC, Pittsburgh, PA USA; 3 Raleigh, NC, USA; 4 Maryland University of Integrative Health, Laurel, MD USA; 5 Schultz Consulting, LLC, Eagan, MN USA; 6 Real Balance Global Wellness, Ft. Collins, CO USA; 7 IHWC Productions, Bloomington, MN USA

**Keywords:** Group coaching, Group health and wellness coaching, Coaching competencies, Group coaching competencies, Validation study

## Abstract

**Background:**

As the popularity and demonstrated effectiveness of Health and Wellness Coaching (HWC) continue to grow to address chronic disease prevalence worldwide, delivery of this approach in a group format is gaining traction, particularly in healthcare. Nonetheless, very little empirical work exists on group coaching and there are currently no published competencies for Group Health and Wellness Coaching (GHWC).

**Methods:**

We used a well-established two-phase (Development and Judgment) process to create and validate GHWC competencies with strong content validity.

**Results:**

Seven highly qualified Subject Matter Experts systematically identified and proposed the GHWC competencies, which were then validated by 78 National Board Certified Health and Wellness Coaches (NBC-HWCs) currently practicing GHWC who rated the importance and use frequency of each one. The validation study led to 72 competencies which are organized into the structure and process of GHWC.

**Conclusions:**

GHWC requires not only coaching skills, but significant group facilitation skills to guide the group process to best support members in maximizing health and well-being through self-directed behavioral change. As the presence of HWC continues to grow, it is imperative that GHWC skill standards be accepted and implemented for the safety of the public, the effectiveness of the intervention, and the value analysis of the field. Such standards will guide curriculum development, allow for a more robust research agenda, and give practical guidance for health and wellness coaches to responsibly run groups. High quality standards for GHWC are particularly needed in health care, where a Level III Current Procedural Terminology (CPT®) code for GHWC has been approved in the United States since 2019 and reimbursement of such has been approved by the Centers for Medicare and Medicaid for 2024.

**Supplementary Information:**

The online version contains supplementary material available at 10.1186/s12913-024-10704-x.

## Background

Health and Wellness Coaching (HWC) is widely accepted as an efficacious intervention to address chronic disease prevalence worldwide [1–5]. HWC can be provided in a group format and is gaining traction as a delivery model in healthcare. In the United States, a Level III Current Procedural Terminology (CPT®) code for group health coaching was approved by the American Medical Association in 2019 [6] and the Centers for Medicare and Medicaid have approved temporary coverage for such, beginning this year (2024) [7]. Nonetheless, very little empirical work exists on group coaching of any kind [8, 9] and no published competencies currently exist for Group Health and Wellness Coaching (GHWC).

As defined by the National Board for Health and Wellness Coaching (NBHWC), GHWC is “a synchronous, facilitated, small (ideally 6 to 12 participants) group process led by a National Board Certified Health and Wellness Coach (NBC-HWC) with the intention of maximizing the combined experience and wisdom of the group to support the achievement of each individual participant’s goals for health and well-being, while optimizing the stability and functioning of the group. Attention to group participants is provided equitably across sessions, with no more than 25% of each session allocated to providing information” [10]. The definition goes on to differentiate GHWC from team coaching, educational groups, fitness groups, conventional group therapy, support groups, and others.

Despite reportedly wide and growing use of group coaching to improve health and well-being, there is shockingly scant empirical work on the topic. There are anecdotal reports and case studies that state the benefits of group coaching [11, 12] and a body of theoretical literature that attempts to clarify the definition of group coaching [8] yet there are few empirical trials of group coaching in any field. Those that exist tend to assess the potential of group coaching to enhance leadership and job performance [13, 14]. We could find only four studies on group coaching for health and well-being; one is a case study [15] and the other three are descriptive rather than experimental [16–18]. There is also a quasi-experimental trial that assessed the impact of a weekly coaching workshop (*n* = 23) versus a control (unstructured discussion group, *n* = 17) [19]. In this trial, attendance for at least 4 of 6 group gatherings was required to be included in the analyses. Compared to the control group, those who attended the life coaching workshops improved more on self-report measures of personal growth, self-acceptance, purpose in life, pleasure and self-efficacy at post-treatment and three months later. Finally, we could only find a single experimental trial on group coaching [20]. This empirical trial was set in an organizational context rather than healthcare, but it did target a health and well-being measure. This randomized controlled trial compared participants assigned to dyadic coaching, group coaching or a control group to assess individual goal attainment and intrinsic motivation related to procrastination [20]. While procrastination improved in all three arms, those in both coaching arms better attained their goals; furthermore, group coaching would likely require fewer resources to meet the company’s needs.

Despite the dearth of efficacy or effectiveness trials, GHWC is rapidly proliferating in many forms across healthcare and industry. HWC delivered in a group format is a potentially cost-effective way to enhance accessibility and empower individuals to make self-directed lifestyle changes, while also offering the potential to leverage community in engaging and motivating participants to improve their overall health and well-being [15–18].

Nonetheless, as industries including healthcare are rapidly adopting processes referred to as “group coaching,” there are myriad types of groups which add further confusion to the field. For example, peer to peer groups (e.g. peer support groups through Veteran’s Affairs) [21], team coaching in the workplace (e.g. for team building and to increase organizational effectiveness), [8] and defined audience educational or support groups [e.g. Alcoholics Anonymous, National Diabetes Prevention Program (NDPP)] are often called group coaching. In fact, the lack of empirical studies on “group coaching” may in part be hampered by the fact that there are diverse definitions for “group coaching” and many include heterogeneous coaching processes as well as content education, training, group facilitation, peer-to-peer coaching, and assessment feedback [8]. The result is a significant need to have a uniform definition and validated GHWC competencies.

The need for standardization of competencies for individual health and wellness coaching similarly emerged about a deacde ago. In 2014, the NBHWC^1^ addressed this need by identifying and validating competencies for individual HWC using a Job Task Analysis and subsequent validation study [22, 23]. The competencies for individual health and wellness coaches became the cornerstone of the Content Outline for the NBHWC certification, the blueprint for the national board examination created by subject matter experts (SMEs) and the National Board of Medical Examiners [24]. To date, over 10,000 coaches have demonstrated the knowledge and practical skills to earn the designation National Board Certified Health and Wellness Coach (NBC-HWC) [25].

Following a comparable path, the NBHWC aims to provide clarification to the definition of GHWC by detailing and validating the competencies needed to perform this role. The creation and validation of competencies is typically demonstrated using a well-established two-phase process that ensures that the competencies have strong content validity [22, 26, 27]. Content validity is the “determination of the content representativeness or content relevance of the elements/items of an instrument by the application of a two-stage (development and judgment) process. When content validity has been viewed as a one-stage process (either development or judgment), it has been challenged most as a form of validity. Using a two-stage process is fundamental to virtually all validation of instrumentation” [26]. This two phase process serves as the industry standard, and more recently, some have separately labeled the implementation of Phase II findings as a third phase [28]. Typically, in Phase I (Development Phase), highly experienced SMEs are selected; it is the consistent and iterative interactions of these SMEs whose consensus-based work produces the Phase I data. This data is then systematically assessed in Phase II (Judgment Phase) by a larger group with relevant experience. The findings from Phase II are then implemented, removing items which do not demonstrate strong content validity.

Such clarification will better advance the use of GHWC in both clinical and community settings, standardize interventions for evaluation in research, and guide curriculum standards for training and professional development. Without definitional clarity on the competencies involved in providing GHWC, rigorous efficacy and effectiveness research are unable to move forward.

## Methods

In Phase I (Development Phase), the NBHWC selected applicant SMEs to appoint a GHWC Task Force which created the proposed list of competencies. In Phase II (Judgment Phase), the proposed GHWC competencies were systematically assessed to validate and finalize the competency list. Each item’s content validity was evaluated on both relevance/importance and frequency of use, as described in detail below.

### Phase I (Development): identifying the competencies

#### Selection of the task force

To recruit potential GHWC SMEs, we used a non-probability, targeted sampling procedure to reach potential SMEs with appropriate expertise to serve on the Task Force. First, the NBHWC appointed two Task Force Leaders, a female MD, NBC-HWC and a male PhD, MCC, NBC-HWC who both had extensive practical experience teaching and facilitating GHWC. Task Force Leaders oversaw the selection of additional SMEs as follows. The NBHWC emailed a request (see Suppl 1) to NBHWC contacts in May 2020, who included all NBHWC-approved and transitionally approved Program Directors, Continuing Education providers, NBC-HWCs who had previously noted interest in group coaching, as well as those in the NBHWC Marketing Outreach data base. The email asked for volunteers who were qualified and willing to serve as SMEs for a GHWC Task Force charged with developing a definitive list of competencies unique to delivering HWC in a group format. Interested volunteers were required to meet the following qualifications:

- Be certified as a NBC-HWC;
- Possess a deep understanding of the knowledge and skills required to facilitate GHWC sessions; and
- Have completed a minimum of 15 h of training in group coaching, or equivalent experience in developing and teaching GHWC curriculum that was based on peer-reviewed references or published books, where they existed.

The email requesting volunteer applicants also asked recipients to pass the invitation to anyone else who might qualify and be interested. The Task Force Leaders then reviewed the pool of applicants, interviewed a subset of these applicants who appeared highly qualified, and then selected SMEs whose schedules aligned. The SMEs were charged with researching, curating, and identifying best practices in the field of GHWC to develop a list of GHWC Competencies.

#### Process of defining the proposed GHWC competencies

The GHWC Task Force built upon the groundwork and collaborations of two prior working groups: one focused on the validated competencies for individual coaches [22, 23]. that serve as the foundation for the national board exam for HWC [29]; and the second focused on establishing group coaching competencies for the Centers for Disease Control and Prevention (CDC) National Diabetes Prevention Program [30]. The GHWC Task Force presumed that group facilitators would be skilled in the validated individual HWC competencies expected of any NBC-HWC, and endeavored to articulate only those aspects that were unique to coaching in a group format.

The GHWC Task Force met virtually over the course of 15 months (June 2020 – October 2021) to engage in a community-based participatory research (CBPR) process that integrated the formative points of view of multiple stakeholders [31]. During Phase I, this CBPR process included the following six steps. First, the SMEs conducted an environmental scan of the group coaching theory and practice literature as well as publications in the commercial GHWC training field to create a curated inventory of scientific literature and innovative practices in the GHWC field. Second, each SME individually reviewed an assigned sub-set of the curated empirical and commercial sources and identified potential GHWC competencies that align with, but do not duplicate, the individual HWC competencies previously defined by NBHWC. Third, the SME Task Force combined the findings of the individual SMEs to compile the literature-based potential competencies into a master draft. Fourth, they conducted a series of review sessions in which the SMEs reviewed the master draft to ensure internal consistency and clarity for the practice field. Fifth, they examined each proposed competency individually to avoid misalignment and potential duplication with the previously established individual HWC competencies. They also revised the potential GHWC competencies to create clear and plain language recommendations. Sixth, the GHWC competency list was proposed to the NBHWC Board of Directors as ready to be validated by a larger audience to further assess content validity and practice field relevance. See Tables 3 & 4 for the proposed specific competencies.

### Phase II (Judgement): validating the GHWC competencies

Four SMEs from the original panel completed Phase I, and an additional four SMEs with GHWC expertise as well as experience with validation studies and manuscript-writing joined for Phase II to continue the CBPR process with three additional steps. First, Phase II experts conducted a validation survey with NBHWC-approved training programs and NBC-HWCs to establish content validity. Second, they calculated task and domain weights using the respondents’ importance and frequency ratings. Third, per industry standards, they removed those competencies that did not meet an Importance score of 3.0 [22, 32, 33]. Phase II processes provided a 'cross-check' of the proposed competencies, to deter potential Task Force bias and to provide stakeholder engagement into the competency development process. In essence, Phase II allowed for a systematic assessment of the proposed competency list.

#### Validation survey

The list of proposed competencies was formatted into a survey (see Suppl 2) and disseminated through SurveyMonkey^®^ software (www.surveymonkey.com) to NBC-HWCs. The survey was structured to ensure that respondents had adequate, recent experience to serve in the validation sample. Hence, at the beginning of the survey the NBHWC definition for GHWC was provided and respondents had to affirm that the groups they provided met the NBHWC definition for GHWC, and that in the prior five years, they had provided at least two group cohorts (series with fixed membership), for at least four synchronous sessions per cohort, or one cohort for ten or more sessions. The survey required 20- 30 min; SurveyMonkey^®^ estimated an average of 23 min. Participants were first asked to rate the importance of each competency in performing GHWC on a four-point Likert scale, with 1 meaning “not important” and 4 meaning “very important.” Then, participants were asked to rate how frequently they used each competency while providing GHWC on a five-point scale, with 1 meaning “never” and 5 meaning “very frequently.”

#### Data analysis

**Fig. 1:**
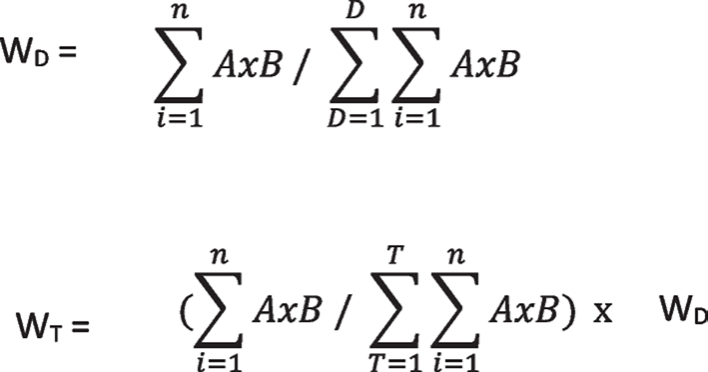
Formulas for domain weights and task weights W_D_ = Domain Weight; W_T_ = Task Weight; A = Importance Rating; B = Frequency Rating; D = Domain; T = Task; and *n* = Number of Responses

Data collected through SurveyMonkey^®^ was exported to EXCEL where descriptive statistics, frequency counts, and task weights were calculated. Task weights using the importance and frequency ratings were determined per industry standards with the formulas in Fig. 1 [22, 32, 33].

## Results

### GHWC task force

Twenty-two SMEs applied to participate in the GHWC Task Force, and in June 2020, five were selected. They joined the two Task Force Leaders from NBHWC, both of whom also met the qualifications. The Task Force SMEs had significant practical experience with GHWC (> 120 years combined), teaching and curriculum development histories (> 75 years combined), and significant familiarity with relevant existing literature. SMEs held degrees that included two PhDs, two EdDs, a MD, a MA, and a RN/BS; two were also trainers for the NDPP. They worked in diverse environments including academia, commercial health coach training programs, and corporate institutions. While sociodemographic information was not systematically collected or used for selection, the Task Force appeared to include six white females and one white male.

### The validation sample

Of the 4770 electronic invitations to the validation survey, 4711 were successfully delivered and 2891 coaches opened the survey. Reminders to complete the survey were sent to the 1820 who had not yet responded. Of the 2891 who opened the invitation, 669 opened the actual survey, and 366 responded in some format, delivering a response rate of 12.7%. Of those who responded, 180 (49%) met the experience eligibility criteria. Of the 180 eligible, 78 (43%) completed all survey items, and thus their responses were utilized to validate the competency list. The Validation Sample’s demographics and coaching practice data are shown in Table 1. Sociodemographic data was requested from the 669 participants who opened the actual survey. Of these, 121 (18%) provided demographics information. Significant diversity was achieved in age and practice settings. Racial and ethnic diversity were limited (92% white and 95% non-Hispanic, Latino/a, or Spanish), as was gender heterogeneity (90% female). The mean Importance and Frequency ratings for each competency, along with task and domain weights are found in Tables 3 and 4.

**Table 1:** Demographics and coaching practice data for subset of subject matter experts who responded to the validation survey (*n* = 121)

	**Frequency n (%)**
**Age range (yrs)**	
20–29	4 (3.3%)
30–39	14 (11.6%)
40–49	37 (30.6%)
50–59	36 (29.8%)
60–69	23 (19.0%)
70 +	7 (5.8%)
**Sex**	
Female	108 (89.3%)
Male	13 (10.7%)
**Race**	
White	111 (91.7%)
Black or African American	6 (5%)
Asian	4 (3.3.%)
American Indian or Alaska Native	3 (2.5%)
Native Hawaiian or Pacific Islander	1 (0.8%)
Other	4 (3.3%)
**Ethnicity**	
Hispanic, Latino/a, Spanish Origin	6 (5.0%)
Non-Hispanic, Latino/a, Spanish Origin	115 (95%)
**Highest Degree Completed**	
Ph.D./Ed.D/Psy.D	10 (8.3%)
Master’s degree	58 (47.9%)
Bachelor’s degree	41 (33.9%)
Associate degree	5 (4.1%)
High School Diploma/GED	7 (5.8%)
**Current Employment as a Health & Wellness Coach**	
Full-Time	60 (49.6%)
Part-Time	61 (50.4%)
**Years Practicing as a Health & Wellness Coach**	
Less than 1	6 (5.0%)
1–2	19 (15.7%)
2–3	33 (27.3%)
4 or more	64 (52.9%)
**Primary Coaching Setting**	
Self-Employed	49 (40.5%)
For-Profit Entity	29 (24.0%)
Medical Setting	22 (19.2%)
Government	20 (16.5%)
Educational Setting	20 (16.5%)
Not-for-Profit Organization	9 (7.4%)
Wellness/Fitness Center	2 (1.6%)
Other	5 (4.1%)

### Proposed GHWC competencies

The GHWC Task Force identified 77 unique GHWC competencies, which were divided into two domains: Coaching Structure and Coaching Process. The Coaching Structure Competencies (Domain 1) and Coaching Process Competencies (Domain 2) were then clusterd into four and five categories, respectively (Table 2).

**Table 2:** Domains and categories of GHWC competencies

**1. Coaching Structure Competencies**
** 1.1** Before First Session
** 1.2** First Session
** 1.3** Structure in All Sessions
** 1.4** Final Session
**2. Coaching Process Competencies: Coaching Relationship/Communication/Techniques**
** 2.1** Client-Centered Relationship
** 2.2** Managing Group Dynamics and Challenges
** 2.3** Model Active, Mindful Listening and Non-Judgmental Presence
** 2.4** Set Goals, Implement Action Commitments, and Review Progress
** 2.5** Enhance Social Support

Weightings for the Coaching Structure domain, divided by categories, are presented in Table 3, along with the relevant individual competencies and their relative weights.

**Table 3:** Domain 1: coaching structure competencies, frequency and importance validation ratings (*N* = 78; Domain weight = 36.3%)

**1.1**	**Before First Session**	IMP	FREQ	WT
1.1.1	Clearly identify initial theme/focus for the offered group and communicate that appropriately in marketing materials	3.65	4.41	0.49
1.1.2	Pre-screen/interview interested group participants individually to: • Explore the opportunities and responsibilities • Confirm commitment of joining a group • Listen for common requests and themes • Answer any individual questions • Note group fit (or not) and identify any need for another type of support • Discuss and administer any assessment tools used	3.37	3.95	0.41
1.1.3	Identify any accommodations needed	3.21	3.77	0.38
1.1.4	Establish all aspects of the Coaching Agreement and send to group members	3.41	4.06	0.43
1.1.5	Create a customized structure for sessions that aligns with group needs	3.56	4.38	0.48
1.1.6	Meet different participant preferences for technology, interactive activities, content sharing, etc	3.05	3.92	0.38
1.1.7	Balance structured activities with space for group processing	3.60	4.51	0.49
1.1.8	Confirm size and make-up of the group to optimize participant experience, for both individual sharing and group connection (recommendation 6–8 optimal, 12 max.)	3.46	4.32	0.46
1.1.9	Manage the room and setup (virtual or in-person) to create an optimal learning environment	3.58	4.47	0.49
1.1.10	If meeting virtually, test the technology, ensure the facilitator(s) have proper equipment and support, and provide participants with instructions for using the technology	3.60	4.28	0.48
1.1.11	Review completed assessments and other data sources	3.42	4.21	0.44
**1.2**	**First Session**			
1.2.1	Set the climate/stage by establishing and maintaining a safe and inclusive group container	3.92	4.63	0.54
1.2.2	Ask for identification preferences such as name, pronoun, etc	3.29	3.77	0.39
1.2.3	Review basic group coaching process	3.44	4.19	0.46
1.2.4	Establish, demonstrate, and maintain agreed upon group guidelines for safety and productivity	3.74	4.42	0.50
1.2.5	Create supportive physical or virtual space	3.65	4.53	0.50
1.2.6	Encourage each participant to take ownership of the process	3.71	4.41	0.49
1.2.7	Discuss communication preferences between sessions	2.95	3.68	0.34
1.2.8	Invite the voluntary participant sharing of contact information between members	2.62	3.24	0.28
**1.3**	**Structure in All Sessions**			
1.3.1	Review larger intention for this group, and (Session 2 and forward) consider themes or needs that arose from the previous session, to set an initial plan for this session	3.45	4.29	0.46
1.3.2	Confirm logistics (meeting location, conference call arrangements, etc.) ensuring all group members are aware and able to access	3.71	4.47	0.51
1.3.3	Provide a flexible agenda or outline in order to best manage time, flow, and focus of session	3.41	4.38	0.46
1.3.4	Invite participants to check-in on state of being, prior session action steps, and needs for the session	3.58	4.40	0.48
1.3.5	Create opportunities for participant interactions with clearly established logistics, guidelines, instructions, and boundaries	3.59	4.45	0.48
1.3.6	End each session with an appropriate closing and check-out	3.68	4.60	0.51
**1.4**	**Final Session**			
1.4.1	Create a closing outline that includes: • Inviting the clients to reflect on, assess, and to articulate progress made, challenges experienced, lessons learned, and growth attained • Finalizing maintenance plans and sustainable pathways forward • Facilitating members in identifying and accessing social supports, services, and other resources	3.88	4.64	0.54
1.4.2	Invite the voluntary participant sharing of contact information between members as the group formally terminates	2.90	3.60	0.34
1.4.3	Formally acknowledge, facilitate celebration of their accomplishments, and close the ‘container’ of the group	3.74	4.60	0.52
1.4.4	When applicable, collect feedback about participant’s group experience	3.65	4.46	0.49

Scores for Importance (IMP) based on 1–4 Likert scale, where: 1 = Not Important; 2 = Somewhat Important; 3 = Important; 4 = Very Important. Scores for Frequency (FREQ) based on 1–5 Likert scale, where: 1 = Never; 2 = Infrequently; 3 = Occasionally; 4 = Frequently; and 5 = Very Frequently

Weightings for the Coaching Process competencies domain, divided by categories are presented in Table 4. Relevant individual competencies and their task weights are also presented.

**Table 4:** Domain 2: coaching process competencies, frequency and importance validation ratings (*N* = 78; Domain weight = 63.7%)

**2.1**	**Client-Centered Relationship**	IMP	FREQ	WT
2.1.1	Continuously recognize the group’s individual and collective needs and design content and activities to meet them	3.59	4.47	0.86
2.1.2	Adjust approach according to the group’s evolving health literacy	3.54	4.28	0.81
2.1.3	Intentionally create a climate that respects social and cultural differences, and fosters inclusivity	3.88	4.56	0.93
2.1.4	Respect and explore the larger meaning of health and wellbeing across diverse group members	3.55	4.22	0.80
**2.2**	**Managing Group Dynamics and Challenges**			
*2.2.1*	*Establish and maintain trust and rapport*			
2.2.2.1	Model unconditional positive regard, benevolence, honesty, sincerity, and authenticity	3.96	4.79	0.99
2.2.1.2	Provide strong leadership and facilitation when the group is forming; respond to the evolving culture and needs of the group	3.82	4.73	0.95
2.2.1.3	Foster shared meaning and honor diversity (e.g. cultural, racial)	3.82	4.44	0.90
2.2.1.4	Monitor appropriate boundaries that meet the needs of individual participants and the group	3.62	4.41	0.86
2.2.1.5	Elicit commitment from participants to attend and to focus their participation by their elimination of all distractions around them	3.53	4.23	0.79
2.2.1.6	Create a comfortable setting, through expressing empathy and friendliness, holding a positive attitude, encouraging participants to share ideas, and building on each participant’s knowledge, as opposed to lecturing	3.95	4.83	1.00
2.2.1.7	Actively care about and equally value each participant with their unique contributions and needs	3.90	4.73	0.97
2.2.1.8	Follow through on commitments made to the group and co facilitators as appropriate	3.90	4.78	0.98
2.2.1.9	Honor participants privacy, confidentiality, choices, expertise and contributions (verbal, written and A/V recordings)	3.95	4.83	1.00
*2.2.2*	*Apply communication skills*			
2.2.2.1	Facilitate development of effective communication skills within the group (e.g. focus on ‘I’ statements, bottom lining/laser speech, and disallowing advice-giving)	3.55	4.23	0.80
2.2.2.2	Elevate the group’s shared wisdom by modeling coaching techniques (e.g. open-ended questions, reflections, affirmations, and intentional silence)	3.77	4.68	0.93
* 2.2.3*	*Provide context and manage participant expectations*			
2.2.3.1	Summarize topics and segue to next topic intentionally	3.41	4.36	0.79
2.2.3.2	Provide instructions for activities, eliciting participant understanding, and clarifying as needed	3.62	4.51	0.86
* 2.2.4*	*Encourage participation and group cohesion*			
2.2.4.1	Identify common themes and link participants into the topic being discussed	3.58	4.47	0.85
2.2.4.2	Harvest group wisdom and resources by identifying themes and new awareness	3.62	4.46	0.86
2.2.4.3	Balance coach guidance with participant and group autonomy	3.67	4.51	0.88
2.2.4.4	Incorporate a range of appropriate group activities, such as open group discussion, round robin, brainstorming, and breakout groups	3.37	4.08	0.74
2.2.4.5	Facilitate a sense of belonging and cultivate interdependence among all group participants	3.63	4.54	0.87
2.2.4.6	Ensure adequate and appropriate attention for each group participant	3.72	4.56	0.90
2.2.4.7	Recognize and create experiences that facilitate discovery while accommodating different learning styles and preferences	3.47	4.22	0.78
2.2.4.8	Promote collaborative discussions by encouraging the acknowledgment of other’s contributions	3.60	4.54	0.87
* 2.2.5*	*Group development and evolution*			
2.2.5.1	Understand and facilitate a participant’s evolution from a self-focus to a collective group focus in regard to goal achievement	2.92	3.71	0.60
2.2.5.2	Recognize that bonding and investment of members builds engagement through the practice of altruism	3.27	3.90	0.69
2.2.5.3	Champion the group by regularly and consistently giving supportive feedback that is specific and timely	3.64	4.55	0.88
2.2.5.4	Understand and facilitate the phases of the group development process	3.32	4.05	0.73
*2.2.6*	*Manage energy, emotions, and flow of the session*	IMP	FREQ	WT
2.2.6.1	Attend to shifts in both individual and group energy (e.g. nonverbal communication signals)	3.60	4.36	0.83
2.2.6.2	Apply nonverbal communication appropriately to the group	3.45	4.24	0.79
2.2.6.3	Demonstrate empathy and honor emotions, recognizing the importance of respecting both individual and group boundaries	3.86	4.71	0.95
2.2.6.4	Manage emotions to create a safe container for the group, by naming and reflecting the emotion	3.69	4.38	0.86
2.2.6.5	Celebrate the forward progress of some, while remaining sensitive to others who may be ‘stuck’	3.74	4.46	0.88
2.2.6.6	Use humor to raise or lighten group energy when it best serves the group process	3.55	4.33	0.81
2.2.6.7	Foster group and individual self-compassion	3.79	4.62	0.92
2.2.7	Manage challenging group participant behaviors (i.e. under or over-talking, interrupting, fixing, arguing, using inappropriate language, being culturally insensitive, veering off topic, etc.) escalating as necessary from general to specific, for example: • Reinforce group honoring of ground rules • Redirect and reframe communication • Openly name and address discord appropriately within the group as it occurs • Have a private discussion with participant(s) outside of group • Attend to patterns of group conflict and resistance • Model skills for conflict management and navigation of difficult conversations	3.78	3.79	0.76
**2.3**	**Model Active, Mindful Listening and Nonjudgmental Presence**			
2.3.1	Take into account differing processing styles and pace	3.76	4.45	0.89
2.3.2	Demonstrate cultural sensitivity and accommodate different world views	3.78	4.18	0.84
**2.4**	**Set Goals, Implement Action Commitments, and Review Progress**			
2.4.1	Consistently integrate goal setting and progress reviews into sessions	3.55	4.41	0.84
2.4.2	Support accountability among group participants	3.47	4.31	0.80
2.4.3	Apply learning to real life goals and action, which may include debriefing of activities	3.67	4.45	0.87
2.4.4	Invite group members to normalize and reframe setbacks, obstacles, and challenges	3.73	4.45	0.87
2.4.5	Honor individual preferences for self-monitoring	3.56	4.23	0.81
2.4.6	Elicit commitment for actions to be taken before the next session	3.51	4.37	0.83
**2.5**	**Enhance Social Support**			
2.5.1	Facilitate participants in developing and accessing social support inside and outside of the group	3.42	4.12	0.76
2.5.2	Bring awareness to outlets participants may utilize outside of sessions, such as social media, to share shifts, breakthroughs, or challenges they experience between sessions	2.94	3.62	0.60
2.5.3	Facilitate participants envisioning how to create needed support outside of the group, transferring the skills they’ve learned in the group	3.42	4.08	0.75

Scores for Importance (IMP) based on 1–4 Likert scale, where: 1 = Not Important; 2 = Somewhat Important; 3 = Important; 4 = Very Important. Scores for Frequency (FREQ) based on 1–5 Likert scale, where: 1 = Never; 2 = Infrequently; 3 = Occasionally; 4 = Frequently; and 5 = Very Frequently

## Discussion

To our knowledge, this is the first published account of a systematic approach to identify and validate the competencies needed for coaches to lead GHWC in a variety of settings. Using well-established industry processes, this competency set is strongly validated. This delineation of group coaching competencies allows for an accurate and valid certification examination that evaluates the examinee's understanding of, and ability to apply the process for GHWC [27, 34]. Further, this validated set of competencies will allow much-needed efficacy and effectivenss research on GHWC to move forward with clearly defined interventions.

Well-accepted criteria for task acceptance includes mean Importance ratings ranging from 2.0 (lenient) to 3.0 (rigorous) [27, 34]. All but five of the 77 proposed competencies achieved a 3.0 or higher and merit inclusion in relevant certification exam specifications. The five that are not included in our finalized set of competencies were mostly related to the degree to which the facilitator(s) promotes the sharing of contact information among group members, openly discusses communication preferences between sessions and promotes resource sharing among participants between sessions. While these five competencies can be used in GHWC, results of the validation study demonstrate they are not necessary. Hence, these competencies should not be expected of all coaches facilitating HWC groups.

The final validated competency set has 72 specific competencies, with 36% falling into the structure domain and 64% falling into the processes domain. Competencies that were most highly weighted (≥ 0.9) tend to relate to the creation of a safe space, respecting individual differences and cultivating an inclusive community, as well as encouraging authenticity and empathy.

The response rate to the validation survey of 12.7% falls within the recommended range of 10–20% [32, 33]. Those who did not complete the survey (79% of those who responded) may not have met the eligibility criteria, as the first part of the survey determined eligibility. While we recognized the potential of lower response rates given the survey design, we determined it most important to include those with adequate and recent experience to provide validation and direction to the current field.

Determination of the number of experts needed in Phase II (Judgment) has always been somewhat arbitrary in judging content validity [26] and many factors contribute to needed sample size [35]. The most important factor in our sample size justification is the universe of potential participants from whom the sample is drawn. This universe is typically assumed to be infinite, but in our study, the sampling universe is actually quite small. There were only 4770 NBC-HWCs at the time of the validation sampling, and only 180 who actually responded and were fully eligible. Of those, 78 (43%) provided complete input, creating a justified sample size. For context, in nursing research, judgment phases rarely require more than 10 experts [26].

The high ratings for content validity are liked related to a fairly homogeneous sample of judges. We weighed the cost versus benefit of having stringent eligibility criteria versus a more diverse participant pool (e.g. from other types of coaching, other credentialling organizations, etc.). However, given the significant dearth of empirical research on efficacy and effectiveness, we chose to keep the eligibility criteria stringent in order to create a more uniform definition of GHWC that would allow the field to move forward from a research perspective. Opening the eligibility criteria significantly would have brought in greater variability in group coaching practices, and contributed even further to the challenging situation regarding a lack of definition for group coaching [9]. It was a judgment call that we carefully made in order to create a clear starting place for GHWC research. Fortunately, the validation sample was diverse in terms of sociodemographics, age and practice settings, lending generalizability to our findings in many respects. While the validation sample was highly educated, largely female, and 92% White, no solid sociodemographic data has been published for the field for comparison.

In the identification of the GWHC competencies, foundational assumptions were made. First and foremost was that these group coaching competencies are built upon the individual HWC competencies previously defined and validated by the NBHWC [22–24], and ideally used by coaches who are board-eligible or NBC-HWCs. Therefore, these competencies articulate only those aspects of expertise that are unique to the group setting, assuming individual coaching competency first.

While differing settings, objectives, and participants may influence the flavor of any particular group coaching offering, the core definition of individual health coaching must hold:

*A patient-centered approach wherein patients at least partially determine their goals, use self-discovery or active learning processes together with content education to work toward their goals, and self-monitor behaviors to increase accountability, all within the context of an interpersonal relationship with a coach. The coach is a health care professional trained in behavior change theory, motivational strategies, and communication techniques, which are used to assist patients to develop intrinsic motivation and obtain skills to create sustainable change for improved health and well-being* [36].

In GHWC sessions, participants often receive brief periods of individualized coaching from the facilitating coach. Such brief, one-on-one encounters provide rich opportunities for other group members through vicarious learning as they discover relatable benefits for their own well-being. In addition, the group members learn to “provide encouragement/affirmation, focus on positive progress, take a nonjudgmental stance throughout the exploration, build rapport, and listen reflectively” [37]. A distinguishing characteristic of GHWC is that group members learn how to effectively support each other using key HWC communication skills as modelled and shaped by the coach. For example, an individual participant is likely to receive genuine affirmations of their efforts, characteristics and skills from other group members rather than, or in addition to, receiving such from the coach facilitator. Similarly, brainstorming potential solutions or next steps for an individual participant in GHWC draws ideas from multiple group members (versus only client and coach) while the individual still makes the autonomous choice of what to try next. Following the coach's model, group members may also learn how to elicit deeper reflection on each others’ actions and choices through the appropriate use of powerful questioning. As a final example, accountability agreements are typically co-created between individual members and their group, building upon the trust and support from within the group. As group members apply their learning from the group experience into their lives, sharing their success and challenges with their peers is especially powerful. These points taken together, the group facilitator not only needs strong individual coaching skills, but needs equally effective group facilitation skills.

We propose that GHWC competencies include the ability to manage group dynamics, build group cohesiveness, facilitate group discussions, provide a psychologically safe environment for all participants, and leverage the power of the group to enhance behavioral change. Hence, we chose to define GHWC as consisting of a committed small group (≤ 12) that meets at least four times. This decision was made to ensure that the competencies could build upon the peer-reviewed literature in terms of what is known about group process. For example, the competencies are designed to parallel the well-established evolution of group dynamics (e.g. forming, storming, norming and performing, [38] with the later addition of adjourning [39]) which have been generalized to coaching [40]. Many factors influence the ideal size of a group [41] and we could find no reference to typical group size in GHWC. Recommendations for size of Motivational Interviewing (MI) groups is 10–12, [42] balancing the need for some education and support. In the organizational development literature, groups sizes of 4–8 are commonly observed, [11, 40] but have also been observed as high as 16 participants [20]. We thus used an evidence-informed approach that values the lived experience and professional perspective of leaders in the field in the early creation of knowledge [43] in deferring to our Phase I SMEs to suggest group size. In GHWC, participants become a member of a type of community, which the coach cultivates to enhance self-directed behavioral change [12]. It is well established that social belonging in and of itself has the power to catalyze meaningful behavior changes [44]. The coach’s skillful use of the group as community is a large part of what differentiates group coaching from other types of groups more typically observed in healthcare such as drop-in support groups and/or education-based programs. While these group types afford individuals the opportunity to form their own supportive connections, they do not intentionally cultivate the group processes that so powerfully impact self-directed health behavior change.

In presenting the competencies for GHWC, we would like to acknowledge the significant overlap in approach with MI in Groups. MI is one of the foundations of HWC [45] and hence MI in Groups is directly applicable. However, MI in Groups often focuses on treatment (e.g. of substance abuse) and is typically led by licensed clinicians [46]. GHWC, on the other hand, often focuses on well-being and lifestyle behaviors and is led by coaches. Treatment group leaders need additional competencies that are not part of coach training. In addition, MI Groups typically draw from Carl Roger’s client-centered therapy process orientation and roughly follow the four phases of MI. When the seminal book on Group MI was written [47] the four MI phases were engagement, exploring perspectives, broadening perspectives, and moving into action. These phases have shifted in the latest edition of MI to engaging, focusing, evoking and planning [46]. Using either flow, GHWC does not necessarily follow these stages, tends to encourage very small steps (“action”) earlier in the process, and uses each step as an experiment from which to learn and discuss with the group.

Group facilitation skills play a seminal role in the GHWC competencies, as they do in other types of groups, including leadership development, team building, addiction recovery, disease management, mental health, peer support, and health education groups [8]. While the GHWC competency set includes the critical shared qualities of leading effective groups in general, it also highlights the knowledge and skills that differentiate it. At the core, GHWC is about enhancing both the group process and coaching skills to assist individuals to maximize their personal health and well-being through self-directed behavioral change. By comparison, in team coaching, the improved functioning of the team for a common purpose is the goal of the intervention [9, 12]. In group leadership coaching, optimizing the ability of an individual to lead teams or organizations is the primary desired outcome and the focus of the coaching [9]. While participant education may play a part in GHWC group, education is not the primary intent. This is in juxtaposition to a group which may use some coaching principles, but which primarily targets the increased knowledge base of the participants. GHWC can integrate an educational section with the topic chosen by the participants, as a way to meet needs and catalyze interest in the group. For individual HWC sessions, keeping the educational portion of each sessionat 25% or less is an NBHWC guideline designed to keep the focus on actual behavioral change [22].

### Strengths and limitations

This delineation of group coaching has both strengths and limitations. First, the decision to build the group competency set on top of individual coaching skills previously defined by the NBHWC allowed for clear focus on how coaches enhance and utilize the group process. However, that decision also limits generalizability to those who have already demonstrated their competency as individual coaches per the national board certification exam. Second, because the demographic and practice data survey in the validation study was sent separately from the validation ratings survey, we can not be sure that the sub-set of individuals who provided their personal data is the same as those who rated the competencies. We do know that 43% of those who opened the actual surveys provided personal data and that 43% of the same pool provided validation ratings. Finally, the limited racial and gender diversity of the original SME panel also limits external validity. That said, this concern is somewhat mitigated by slightly better diversity in the larger validation group, but would best be repeated in a more heterogeneous sample. It is not clear however, what would be a representative sample from the overall field of HWC.

## Conclusion

As the number and presence of NBC-HWC’s continues to grow (i.e. > 10,000 in February 2024), it is critical for the safety of the public, the effectiveness of the intervention, and the value analysis of the field, that GHWC skill standards be accepted and implemented. This is particularly true in health care settings, where Level III CPT® reimbursement codes for group coaching have already been approved [6] and the Centers for Medicare and Medicaid has announced it willreimburse for coaching groups beginning this year (2024) [7]. Given the unlicensed nature of health coaching, it is imperative that consistent standards be established within the field by national leaders, with the engagement of coaches already leading such groups. We intend that these competencies be used to guide the development of curriculum in training programs and in continuing education offerings for HWC; provide some practice standards for any research studies utilizing GHWC as an intervention; and give guidance to HWCs employed in the field as to what competencies they should possess in order to responsibly and effectively run groups. We anticipate that, in the near future, the NBHWC will formally implement processes for recognizing these GHWC competencies in NBC-HWC credentialling.

## Supplementary Information

**Supplementary Material 1.**

**Supplementary Material 2.**

## Data Availability

The Phase I SME application and Phase II validation survey are provided as online supplements. We would be happy to make our validation data available.

## Abbreviations

HWC: Health and wellness coaching
GHWC: Group health and wellness coaching
NBC-HWC: National Board Certified Health and Wellness Coach
NBHWC: National Board for Health and Wellness Coaching
SME: Subject matter expert

## Footnotes

1 Formerly named the National Consortium for Credentialling Health and Wellness Coaches (NCCHWC).
