# Supplementary material for: Group Health & Wellness Coaching: development and validation of the required competencies
Source: BMC Health Serv Res. 2024 Mar 28;24:392. doi: 10.1186/s12913-024-10704-x (PMC10976677; doi:10.1186/s12913-024-10704-x)
Supplement: Supplementary file 1 — Supplementary Material 1. [file 12913_2024_10704_MOESM1_ESM.pdf]

# Group Coaching Task Force

The Group Health and Wellness Coaching (GHWC) Task Force will be composed of 5 to 6 thought leaders in the field, with group coaching teaching and curriculum development experience.

We are looking to develop a team with the following qualifications:

- Must be a National Board Certified Health & Wellness Coach.

- Possess a deep understanding of the knowledge and skills required to facilitate group health & wellness coaching sessions.

- Must have completed a minimum of 15 hours of training, both synchronous and asynchronous in group coaching, or have experience in developing and teaching programs/courses of equal or greater intensity. Developed curricula should have been based on published references, where they exist.

We anticipate that this task force will meet 1-2 times in June with increased meetings if needed in July.  We recognize the multiple demands being made on individuals at this time, and know that it may be a very busy time, especially for educators. We hope that each member will volunteer approximately 10 hours each month, but realize that may need to vary week by week.

* Indicates required question

1. Email Address *

2. First Name *

3. Last Name *

4. Program Completed: *

______________________________

5. Program of Employment *

______________________________

6. Role in the Group Coaching Task Force *

*Check all that apply.*

- [ ] I am interested in being a part of the Group Coaching Task Force.
- [ ] I would like to share a reference.

7. Please share your Group Coaching Training experience.

______________________________
______________________________
______________________________
______________________________
______________________________

8. If you have noted that you have a group coaching reference to share, please upload it here.

______________________________
______________________________
______________________________
______________________________
______________________________

9. Please list any questions you have here.

This content is neither created nor endorsed by Google.

Google Forms
