# Supplementary material for: Group Health & Wellness Coaching: development and validation of the required competencies
Source: BMC Health Serv Res. 2024 Mar 28;24:392. doi: 10.1186/s12913-024-10704-x (PMC10976677; doi:10.1186/s12913-024-10704-x)
Supplement: Supplementary file 2 — Supplementary Material 2. [file 12913_2024_10704_MOESM2_ESM.pdf]

Group Health & Wellness Coaching Validation Survey

Dear Health and Wellness Coach,

The NBHWC is actively working on efforts to improve and advance the field of Health and Wellness coaching, and are now working to establish standards and competencies for Group Health & Wellness Coaching. A national committee of group HWC leaders and educators of diverse coach training and professional backgrounds have created this competency list, which has been reviewed by the NBHWC Board of Directors. Through this survey, we seek to validate those competencies based upon the experiences of NBC-HWC's with recent or current HWC group coaching experience. If that describes you, we're requesting your participation in the validation process by completing this survey. Your experience and wisdom truly counts in this important endeavor.

This survey will take about 30 minutes to complete. Please use the button below to complete the validation survey by May 16, 2021. Thank you for your valued input.

NBHWC Board & NBHWC Group Coaching Committee

Group Health & Wellness Coaching Validation Survey

Definition

"As defined by the NBHWC, group health and wellness coaching is a synchronous, facilitated, small (ideally 6 to 12 participant) group process led by a National Board Certified Health & Wellness Coach (NBC-HWC) with the intention of maximizing the combined experience and wisdom of the group to support the achievement of each individual participant's goals for health and wellbeing, while optimizing the stability and functioning of the group. Attention to group participants is provided equitably across sessions; with no more than 25% of each session allocated to providing information."

Group Health Coaching is differentiated from, for example:

- Team Coaching is the art of facilitating a real team (e.g. workplace, athletic...) to maximize its performance and enjoyment in service of meaningful organizational goals; essentially the team is the client.

- Educational groups, where more than 25% of the time is spent in teaching information or skills to participants. In education groups, the facilitator sets the agenda/curriculum. This would include programs where the expertise of the facilitators is primary.

- Fitness groups, where the primary goal is acquiring physical activity skills, routines, or encouragement.

- Other such groups may include the traditional delivery of support groups, recovery groups, therapy groups, etc.

For the purposes of these competencies, it is critical to clearly define health coaching groups as consisting of 4 or more participants, which meet live or synchronously for a minimum of 4 sessions longitudinally. Coaches should have run at least 2 such groups, or 1 committed group for 10 or more sessions.

Qualifications to complete

Group Coaching – In order to participate in the validation survey, participants must meet the following requirements:

* I hold an active NBC-HWC credential

◯ Yes

◯ No

NBHWC

Group Health & Wellness Coaching Validation Survey

* Since January 2016, have you provided at least 2 group cohorts, for at least synchronous 4 sessions each, or one cohort for 10 or more sessions, that meet the above definition for group coaching?

◯ Yes

◯ No

Group Health & Wellness Coaching Validation Survey

* The group health & wellness coaching I am referring to with regard to this survey fully meets the definition of group HWC as stated above.

◯ Yes

◯ No

Group Health & Wellness Coaching Validation Survey

Importance and Frequency Scale

Please use the following scales to determine the importance and frequency of each competency listed.

In reflecting on your group HWC coaching experience as reported earlier in this survey, how **IMPORTANT** was each competency?"

The response options for importance are:

Not Important

Somewhat Important

Important

Very Important

In reflecting on your group HWC coaching experience as reported earlier in this survey, how **FREQUENTLY** was each competency performed?

The response options for frequency are:

Never

Infrequently

Occasionally

Frequently

Very frequently

Group Health & Wellness Coaching Validation Survey

1. Coaching Structure Competencies

1.1 Before the First Session

Overview: Before the first group session, the facilitating coach(es) assures the fit and commitment of each member for the planned group, answers any questions, and confirms dates, times and location or virtual process for the sessions.

1.1.1 Clearly identify initial theme/focus for the offered group and communicate that appropriately in marketing materials

* In reflecting on your group Health and Wellness coaching experience as reported earlier in this survey, how IMPORTANT was this competency?

The response options for importance are:

- ◯ Not Important
- ◯ Somewhat Important
- ◯ Important
- ◯ Very Important

* In reflecting on your group Health and Wellness coaching experience as reported earlier in this survey, how FREQUENTLY was this competency performed?

The response options for frequency are:

- ◯ Never
- ◯ Infrequently
- ◯ Occasionally
- ◯ Frequently
- ◯ Very frequently

1.1.2 Pre-screen/interview interested group participants individually to:

- **Explore the opportunities and responsibilities**
- **Confirm commitment of joining a group**
- **Listen for common requests and themes**
- **Answer any individual questions**
- **Note group fit (or not) and identify any need for another type of support**
- **Discuss and administer any assessment tools used**

* In reflecting on your group Health and Wellness coaching experience as reported earlier in this survey, how IMPORTANT was this competency?

The response options for importance are:

- ◯ Not Important
- ◯ Somewhat Important
- ◯ Important
- ◯ Very Important

* In reflecting on your group Health and Wellness coaching experience as reported earlier in this survey, how FREQUENTLY was this competency performed?

The response options for frequency are:

◯ Never

◯ Infrequently

◯ Occasionally

◯ Frequently

◯ Very frequently

1.1.3 Identify any accommodations needed

* In reflecting on your group Health and Wellness coaching experience as reported earlier in this survey, how IMPORTANT was this competency?

The response options for importance are:

◯ Not Important

◯ Somewhat Important

◯ Important

◯ Very Important

* In reflecting on your group Health and Wellness coaching experience as reported earlier in this survey, how FREQUENTLY was this competency performed?

The response options for frequency are:

◯ Never

◯ Infrequently

◯ Occasionally

◯ Frequently

◯ Very frequently

1.1.4 Establish all aspects of the Coaching Agreement and send to group members

* In reflecting on your group Health and Wellness coaching experience as reported earlier in this survey, how IMPORTANT was this competency?

The response options for importance are:

◯ Not Important

◯ Somewhat Important

◯ Important

◯ Very Important

* In reflecting on your group Health and Wellness coaching experience as reported earlier in this survey, how FREQUENTLY was this competency performed?

The response options for frequency are:

◯ Never

◯ Infrequently

◯ Occasionally

◯ Frequently

◯ Very frequently

1.1.5 Create a customized structure for sessions that aligns with group needs

* In reflecting on your group Health and Wellness coaching experience as reported earlier in this survey, how IMPORTANT was this competency?

The response options for importance are:

◯ Not Important

◯ Somewhat Important

◯ Important

◯ Very Important

* In reflecting on your group Health and Wellness coaching experience as reported earlier in this survey, how FREQUENTLY was this competency performed?

The response options for frequency are:

- Never
- Infrequently
- Occasionally
- Frequently
- Very frequently

1.1.6 Meet different participant preferences for technology, interactive activities, content sharing, etc.

* In reflecting on your group Health and Wellness coaching experience as reported earlier in this survey, how IMPORTANT was this competency?

The response options for importance are:

- Not Important
- Somewhat Important
- Important
- Very Important

* In reflecting on your group Health and Wellness coaching experience as reported earlier in this survey, how FREQUENTLY was this competency performed?

The response options for frequency are:

- Never
- Infrequently
- Occasionally
- Frequently
- Very frequently

1.1.7 Balance structured activities with space for group processing

* In reflecting on your group Health and Wellness coaching experience as reported earlier in this survey, how IMPORTANT was this competency?

The response options for importance are:

- ◯ Not Important
- ◯ Somewhat Important
- ◯ Important
- ◯ Very Important

* In reflecting on your group Health and Wellness coaching experience as reported earlier in this survey, how FREQUENTLY was this competency performed?

The response options for frequency are:

- ◯ Never
- ◯ Infrequently
- ◯ Occasionally
- ◯ Frequently
- ◯ Very frequently

1.1.8 Confirm size and make-up of the group to optimize participant experience, for both individual sharing and group connection (recommendation 6-8 optimal, 12 max.)

* In reflecting on your group Health and Wellness coaching experience as reported earlier in this survey, how IMPORTANT was this competency?

The response options for importance are:

- ◯ Not Important
- ◯ Somewhat Important
- ◯ Important
- ◯ Very Important

* In reflecting on your group Health and Wellness coaching experience as reported earlier in this survey, how FREQUENTLY was this competency performed?

The response options for frequency are:

- Never
- Infrequently
- Occasionally
- Frequently
- Very frequently

1.1.9 Manage the room and setup (virtual or in-person) to create an optimal learning environment

* In reflecting on your group Health and Wellness coaching experience as reported earlier in this survey, how IMPORTANT was this competency?

The response options for importance are:

- Not Important
- Somewhat Important
- Important
- Very Important

* In reflecting on your group Health and Wellness coaching experience as reported earlier in this survey, how FREQUENTLY was this competency performed?

The response options for frequency are:

- Never
- Infrequently
- Occasionally
- Frequently
- Very frequently

1.1.10 If meeting virtually, test the technology, ensure the facilitator(s) have proper equipment and support, and provide participants with instructions for using the technology

* In reflecting on your group Health and Wellness coaching experience as reported earlier in this survey, how IMPORTANT was this competency?

The response options for importance are:

- ◯ Not Important
- ◯ Somewhat Important
- ◯ Important
- ◯ Very Important

* In reflecting on your group Health and Wellness coaching experience as reported earlier in this survey, how FREQUENTLY was this competency performed?

The response options for frequency are:

- ◯ Never
- ◯ Infrequently
- ◯ Occasionally
- ◯ Frequently
- ◯ Very frequently

1.1.11 Review completed assessments and other data sources

* In reflecting on your group Health and Wellness coaching experience as reported earlier in this survey, how IMPORTANT was this competency?

The response options for importance are:

- ◯ Not Important
- ◯ Somewhat Important
- ◯ Important
- ◯ Very Important

* In reflecting on your group Health and Wellness coaching experience as reported earlier in this survey, how FREQUENTLY was this competency performed?

The response options for frequency are:

- ◯ Never
- ◯ Infrequently
- ◯ Occasionally
- ◯ Frequently
- ◯ Very frequently

Group Health & Wellness Coaching Validation Survey

1.1 First Session

Overview: The coach's aims for the initial session are to describe the group coaching process, establish group agreements, clarify roles and expectations for the process for both the participants and the facilitator(s).

1.2.1 Set the climate/stage by establishing and maintaining a safe and inclusive group container

* In reflecting on your group Health and Wellness coaching experience as reported earlier in this survey, how IMPORTANT was this competency?

The response options for importance are:

- ◯ Not Important
- ◯ Somewhat Important
- ◯ Important
- ◯ Very Important

* In reflecting on your group Health and Wellness coaching experience as reported earlier in this survey, how FREQUENTLY was this competency performed?

The response options for frequency are:

- Never
- Infrequently
- Occasionally
- Frequently
- Very frequently

1.2.2 Ask for identification preferences such as name, pronoun, etc.

* In reflecting on your group Health and Wellness coaching experience as reported earlier in this survey, how IMPORTANT was this competency?

The response options for importance are:

- Not Important
- Somewhat Important
- Important
- Very Important

* In reflecting on your group Health and Wellness coaching experience as reported earlier in this survey, how FREQUENTLY was this competency performed?

The response options for frequency are:

- Never
- Infrequently
- Occasionally
- Frequently
- Very frequently

1.2.3 Review basic group coaching process

* In reflecting on your group Health and Wellness coaching experience as reported earlier in this survey, how IMPORTANT was this competency?

The response options for importance are:

- Not Important
- Somewhat Important
- Important
- Very Important

* In reflecting on your group Health and Wellness coaching experience as reported earlier in this survey, how FREQUENTLY was this competency performed?

The response options for frequency are:

- Never
- Infrequently
- Occasionally
- Frequently
- Very frequently

1.2.4 Establish, demonstrate, and maintain agreed upon group guidelines for safety and productivity

* In reflecting on your group Health and Wellness coaching experience as reported earlier in this survey, how IMPORTANT was this competency?

The response options for importance are:

- Not Important
- Somewhat Important
- Important
- Very Important

* In reflecting on your group Health and Wellness coaching experience as reported earlier in this survey, how FREQUENTLY was this competency performed?

The response options for frequency are:

- ◯ Never
- ◯ Infrequently
- ◯ Occasionally
- ◯ Frequently
- ◯ Very frequently

1.2.5 Create supportive physical or virtual space

* In reflecting on your group Health and Wellness coaching experience as reported earlier in this survey, how IMPORTANT was this competency?

The response options for importance are:

- ◯ Not Important
- ◯ Somewhat Important
- ◯ Important
- ◯ Very Important

* In reflecting on your group Health and Wellness coaching experience as reported earlier in this survey, how FREQUENTLY was this competency performed?

The response options for frequency are:

- ◯ Never
- ◯ Infrequently
- ◯ Occasionally
- ◯ Frequently
- ◯ Very frequently

1.2.6 Encourage each participant to take ownership of the process

* In reflecting on your group Health and Wellness coaching experience as reported earlier in this survey, how IMPORTANT was this competency?

The response options for importance are:

- ◯ Not Important
- ◯ Somewhat Important
- ◯ Important
- ◯ Very Important

* In reflecting on your group Health and Wellness coaching experience as reported earlier in this survey, how FREQUENTLY was this competency performed?

The response options for frequency are:

- ◯ Never
- ◯ Infrequently
- ◯ Occasionally
- ◯ Frequently
- ◯ Very frequently

1.2.7 Discuss communication preferences between sessions

* In reflecting on your group Health and Wellness coaching experience as reported earlier in this survey, how IMPORTANT was this competency?

The response options for importance are:

- ◯ Not Important
- ◯ Somewhat Important
- ◯ Important
- ◯ Very Important

* In reflecting on your group Health and Wellness coaching experience as reported earlier in this survey, how FREQUENTLY was this competency performed?

The response options for frequency are:

- Never
- Infrequently
- Occasionally
- Frequently
- Very frequently

1.2.8 Invite the voluntary participant sharing of contact information between members

* In reflecting on your group Health and Wellness coaching experience as reported earlier in this survey, how IMPORTANT was this competency?

The response options for importance are:

- Not Important
- Somewhat Important
- Important
- Very Important

* In reflecting on your group Health and Wellness coaching experience as reported earlier in this survey, how FREQUENTLY was this competency performed?

The response options for frequency are:

- Never
- Infrequently
- Occasionally
- Frequently
- Very frequently

Group Health & Wellness Coaching Validation Survey

1.3 Structure in all sessions

Overview: Group coaching sessions are usually planned for a predetermined period of weeks or months, at a preset length and frequency. At the opening of each session, the coach routinely leads a check-in/self-assessment activity for participants and establishes a focus for the session. Using coaching processes as appropriate, coach(es) support the participants in recognizing new awarenesses, gaining new skills, and potentially defining new action steps.

1.3.1 Review larger intention for this group, and (session 2 and forward) consider themes or needs that arose from the previous session, to set an initial plan for this session

* In reflecting on your group Health and Wellness coaching experience as reported earlier in this survey, how IMPORTANT was this competency?

The response options for importance are:

◯ Not Important

◯ Somewhat Important

◯ Important

◯ Very Important

* In reflecting on your group Health and Wellness coaching experience as reported earlier in this survey, how FREQUENTLY was this competency performed?

The response options for frequency are:

◯ Never

◯ Infrequently

◯ Occasionally

◯ Frequently

◯ Very frequently

1.3.2 Confirm logistics (meeting location, conference call arrangements, etc.) ensuring all group members are aware and able to access

* In reflecting on your group Health and Wellness coaching experience as reported earlier in this survey, how IMPORTANT was this competency?

The response options for importance are:

- ◯ Not Important
- ◯ Somewhat Important
- ◯ Important
- ◯ Very Important

* In reflecting on your group Health and Wellness coaching experience as reported earlier in this survey, how FREQUENTLY was this competency performed?

The response options for frequency are:

- ◯ Never
- ◯ Infrequently
- ◯ Occasionally
- ◯ Frequently
- ◯ Very frequently

1.3.3 Provide a flexible agenda or outline in order to best manage time, flow, and focus of session

* In reflecting on your group Health and Wellness coaching experience as reported earlier in this survey, how IMPORTANT was this competency?

The response options for importance are:

- ◯ Not Important
- ◯ Somewhat Important
- ◯ Important
- ◯ Very Important

* In reflecting on your group Health and Wellness coaching experience as reported earlier in this survey, how FREQUENTLY was this competency performed?

The response options for frequency are:

- Never
- Infrequently
- Occasionally
- Frequently
- Very frequently

1.3.4 Invite participants to check-in on state of being, prior session action steps, and needs for the session

* In reflecting on your group Health and Wellness coaching experience as reported earlier in this survey, how IMPORTANT was this competency?

The response options for importance are:

- Not Important
- Somewhat Important
- Important
- Very Important

* In reflecting on your group Health and Wellness coaching experience as reported earlier in this survey, how FREQUENTLY was this competency performed?

The response options for frequency are:

- Never
- Infrequently
- Occasionally
- Frequently
- Very frequently

1.3.5 Create opportunities for participant interactions with clearly established logistics, guidelines, instructions, and boundaries

* In reflecting on your group Health and Wellness coaching experience as reported earlier in this survey, how IMPORTANT was this competency?

The response options for importance are:

- ◯ Not Important
- ◯ Somewhat Important
- ◯ Important
- ◯ Very Important

* In reflecting on your group Health and Wellness coaching experience as reported earlier in this survey, how FREQUENTLY was this competency performed?

The response options for frequency are:

- ◯ Never
- ◯ Infrequently
- ◯ Occasionally
- ◯ Frequently
- ◯ Very frequently

1.3.6 End each session with an appropriate closing and check-out

* In reflecting on your group Health and Wellness coaching experience as reported earlier in this survey, how IMPORTANT was this competency?

The response options for importance are:

- ◯ Not Important
- ◯ Somewhat Important
- ◯ Important
- ◯ Very Important

* In reflecting on your group Health and Wellness coaching experience as reported earlier in this survey, how FREQUENTLY was this competency performed?

The response options for frequency are:

◯ Never

◯ Infrequently

◯ Occasionally

◯ Frequently

◯ Very frequently

Group Health & Wellness Coaching Validation Survey

1.4 Final session

Overview: In the final group coaching session, the coach's focus is on recognition of progress, learning, and closure. The participants may be invited to share successes and reflect back at what s/he has learned. The coach(es) invite participants to establish a plan for how they will maintain or continue progressing toward goals, with an emphasis on desired support and resources. Continuing connections are discussed.

1.4.1 Create a closing outline that includes:

- **Inviting the clients to reflect on, assess, and to articulate progress made, challenges experienced, lessons learned, and growth attained**
- **Finalizing maintenance plans and sustainable pathways forward**
- **Facilitating members in identifying and accessing social supports, services, and other resources**

* In reflecting on your group Health and Wellness coaching experience as reported earlier in this survey, how IMPORTANT was this competency?

The response options for importance are:

◯ Not Important

◯ Somewhat Important

◯ Important

◯ Very Important

* In reflecting on your group Health and Wellness coaching experience as reported earlier in this survey, how FREQUENTLY was this competency performed?

The response options for frequency are:

◯ Never

◯ Infrequently

◯ Occasionally

◯ Frequently

◯ Very frequently

1.4.2 Invite the voluntary participant sharing of contact information between members as the group formally terminates

* In reflecting on your group Health and Wellness coaching experience as reported earlier in this survey, how IMPORTANT was this competency?

The response options for importance are:

◯ Not Important

◯ Somewhat Important

◯ Important

◯ Very Important

* In reflecting on your group Health and Wellness coaching experience as reported earlier in this survey, how FREQUENTLY was this competency performed?

The response options for frequency are:

- ◯ Never
- ◯ Infrequently
- ◯ Occasionally
- ◯ Frequently
- ◯ Very frequently

1.4.3 Formally acknowledge, facilitate celebration of their accomplishments, and close the 'container' of the group

* In reflecting on your group Health and Wellness coaching experience as reported earlier in this survey, how IMPORTANT was this competency?

The response options for importance are:

- ◯ Not Important
- ◯ Somewhat Important
- ◯ Important
- ◯ Very Important

* In reflecting on your group Health and Wellness coaching experience as reported earlier in this survey, how FREQUENTLY was this competency performed?

The response options for frequency are:

- ◯ Never
- ◯ Infrequently
- ◯ Occasionally
- ◯ Frequently
- ◯ Very frequently

1.4.4 When applicable, collect feedback about participant's group experience

* In reflecting on your group Health and Wellness coaching experience as reported earlier in this survey, how IMPORTANT was this competency?

The response options for importance are:

- ◯ Not Important
- ◯ Somewhat Important
- ◯ Important
- ◯ Very Important

* In reflecting on your group Health and Wellness coaching experience as reported earlier in this survey, how FREQUENTLY was this competency performed?

The response options for frequency are:

- ◯ Never
- ◯ Infrequently
- ◯ Occasionally
- ◯ Frequently
- ◯ Very frequently

NBHWC

Group Health & Wellness Coaching Validation Survey

2. Coaching Process Competencies: Coaching Relationship/Communication/Techniques

2.1 Client-centered relationship

Overview: A coach facilitates behavior change by empowering participants to self-discover values, resources, and strategies that are individualized and meaningful. In a group, the coach facilitates the interactions between group members, ensuring that the climate of the coaching process is maintained, inviting participants to speak from their own perspective and experience, listen without interruption, and not offer unsolicited opinions or advice. The coach provides the structure of the session, and may provide some educational content or tools/exercises for exploration, but does not serve in a primary role of a content expert or educator.

2.1.1 Continuously recognize the group's individual and collective needs and design content and activities to meet them

* In reflecting on your group Health and Wellness coaching experience as reported earlier in this survey, how IMPORTANT was this competency?

The response options for importance are:

◯ Not Important

◯ Somewhat Important

◯ Important

◯ Very Important

* In reflecting on your group Health and Wellness coaching experience as reported earlier in this survey, how FREQUENTLY was this competency performed?

The response options for frequency are:

◯ Never

◯ Infrequently

◯ Occasionally

◯ Frequently

◯ Very frequently

2.1.2 Adjust approach according to the group's evolving health literacy

* In reflecting on your group Health and Wellness coaching experience as reported earlier in this survey, how IMPORTANT was this competency?

The response options for importance are:

- ◯ Not Important
- ◯ Somewhat Important
- ◯ Important
- ◯ Very Important

* In reflecting on your group Health and Wellness coaching experience as reported earlier in this survey, how FREQUENTLY was this competency performed?

The response options for frequency are:

- ◯ Never
- ◯ Infrequently
- ◯ Occasionally
- ◯ Frequently
- ◯ Very frequently

2.1.3 Intentionally create a climate that respects social and cultural differences, and fosters inclusivity

* In reflecting on your group Health and Wellness coaching experience as reported earlier in this survey, how IMPORTANT was this competency?

The response options for importance are:

- ◯ Not Important
- ◯ Somewhat Important
- ◯ Important
- ◯ Very Important

* In reflecting on your group Health and Wellness coaching experience as reported earlier in this survey, how FREQUENTLY was this competency performed?

The response options for frequency are:

- Never
- Infrequently
- Occasionally
- Frequently
- Very frequently

2.1.4 Respect and explore the larger meaning of health and wellbeing across diverse group members

* In reflecting on your group Health and Wellness coaching experience as reported earlier in this survey, how IMPORTANT was this competency?

The response options for importance are:

- Not Important
- Somewhat Important
- Important
- Very Important

* In reflecting on your group Health and Wellness coaching experience as reported earlier in this survey, how FREQUENTLY was this competency performed?

The response options for frequency are:

- Never
- Infrequently
- Occasionally
- Frequently
- Very frequently

Group Health & Wellness Coaching Validation Survey

2.2 Managing group dynamics and challenges

2.2.1 Establish and maintain trust and rapport

Overview: The coach(es) establishes a positive and safe environment where all participants can feel accepted and supported. Individuals are better able to clarify values and access motivation when they feel safe to be honest and vulnerable. This requires the coach to manage the interpersonal dynamics of the group, including holding individuals to the established group agreements. The coach builds trust by attending to the participants' emotions, as well as words and behaviors. For example, when the coach senses conflict, discomfort or confusion, the coach acknowledges what is happening, and addresses issues appropriately to optimize the safety and functioning of the group.

2.2.1.1 Model unconditional positive regard, benevolence, honesty, sincerity, and authenticity

* In reflecting on your group Health and Wellness coaching experience as reported earlier in this survey, how IMPORTANT was this competency?

The response options for importance are:

- ◯ Not Important
- ◯ Somewhat Important
- ◯ Important
- ◯ Very Important

* In reflecting on your group Health and Wellness coaching experience as reported earlier in this survey, how FREQUENTLY was this competency performed?

The response options for frequency are:

- ◯ Never
- ◯ Infrequently
- ◯ Occasionally
- ◯ Frequently
- ◯ Very frequently

2.2.1.2 Provide strong leadership and facilitation when the group is forming, and respond to the evolving culture and needs of the group

* In reflecting on your group Health and Wellness coaching experience as reported earlier in this survey, how IMPORTANT was this competency?

The response options for importance are:

- ◯ Not Important
- ◯ Somewhat Important
- ◯ Important
- ◯ Very Important

* In reflecting on your group Health and Wellness coaching experience as reported earlier in this survey, how FREQUENTLY was this competency performed?

The response options for frequency are:

- ◯ Never
- ◯ Infrequently
- ◯ Occasionally
- ◯ Frequently
- ◯ Very frequently

2.2.1.3 Foster shared meaning and honor diversity (e.g. cultural, racial)

* In reflecting on your group Health and Wellness coaching experience as reported earlier in this survey, how IMPORTANT was this competency?

The response options for importance are:

- ◯ Not Important
- ◯ Somewhat Important
- ◯ Important
- ◯ Very Important

* In reflecting on your group Health and Wellness coaching experience as reported earlier in this survey, how FREQUENTLY was this competency performed?

The response options for frequency are:

- ◯ Never
- ◯ Infrequently
- ◯ Occasionally
- ◯ Frequently
- ◯ Very frequently

2.2.1.4 Monitor appropriate boundaries that meet the needs of individual participants and the group

* In reflecting on your group Health and Wellness coaching experience as reported earlier in this survey, how IMPORTANT was this competency?

The response options for importance are:

- ◯ Not Important
- ◯ Somewhat Important
- ◯ Important
- ◯ Very Important

* In reflecting on your group Health and Wellness coaching experience as reported earlier in this survey, how FREQUENTLY was this competency performed?

The response options for frequency are:

- ◯ Never
- ◯ Infrequently
- ◯ Occasionally
- ◯ Frequently
- ◯ Very frequently

2.2.1.5 Elicit commitment from participants to attend and to focus their participation by their elimination of all distractions around them

* In reflecting on your group Health and Wellness coaching experience as reported earlier in this survey, how IMPORTANT was this competency?

The response options for importance are:

- ◯ Not Important
- ◯ Somewhat Important
- ◯ Important
- ◯ Very Important

* In reflecting on your group Health and Wellness coaching experience as reported earlier in this survey, how FREQUENTLY was this competency performed?

The response options for frequency are:

- ◯ Never
- ◯ Infrequently
- ◯ Occasionally
- ◯ Frequently
- ◯ Very frequently

2.2.1.6 Create a comfortable setting, through expressing empathy and friendliness, holding a positive attitude, encouraging participants to share ideas, and building on each participant's knowledge, as opposed to lecturing

* In reflecting on your group Health and Wellness coaching experience as reported earlier in this survey, how IMPORTANT was this competency?

The response options for importance are:

- ◯ Not Important
- ◯ Somewhat Important
- ◯ Important
- ◯ Very Important

* In reflecting on your group Health and Wellness coaching experience as reported earlier in this survey, how FREQUENTLY was this competency performed?

The response options for frequency are:

- ◯ Never
- ◯ Infrequently
- ◯ Occasionally
- ◯ Frequently
- ◯ Very frequently

2.2.1.7 Actively care about and equally value each participant with their unique contributions and needs

* In reflecting on your group Health and Wellness coaching experience as reported earlier in this survey, how IMPORTANT was this competency?

The response options for importance are:

- ◯ Not Important
- ◯ Somewhat Important
- ◯ Important
- ◯ Very Important

* In reflecting on your group Health and Wellness coaching experience as reported earlier in this survey, how FREQUENTLY was this competency performed?

The response options for frequency are:

- ◯ Never
- ◯ Infrequently
- ◯ Occasionally
- ◯ Frequently
- ◯ Very frequently

2.2.1.8 Follow through on commitments made to the group and co facilitators as appropriate

* In reflecting on your group Health and Wellness coaching experience as reported earlier in this survey, how IMPORTANT was this competency?

The response options for importance are:

- ◯ Not Important
- ◯ Somewhat Important
- ◯ Important
- ◯ Very Important

* In reflecting on your group Health and Wellness coaching experience as reported earlier in this survey, how FREQUENTLY was this competency performed?

The response options for frequency are:

◯ Never

◯ Infrequently

◯ Occasionally

◯ Frequently

◯ Very frequently

2.2.1.9 Honor participants privacy, confidentiality, choices, expertise and contributions (verbal, written and A/V recordings)

* In reflecting on your group Health and Wellness coaching experience as reported earlier in this survey, how IMPORTANT was this competency?

The response options for importance are:

◯ Not Important

◯ Somewhat Important

◯ Important

◯ Very Important

* In reflecting on your group Health and Wellness coaching experience as reported earlier in this survey, how FREQUENTLY was this competency performed?

The response options for frequency are:

◯ Never

◯ Infrequently

◯ Occasionally

◯ Frequently

◯ Very frequently

Group Health & Wellness Coaching Validation Survey

2.2.2 Apply communication skills

2.2.2.1 Facilitate development of effective communication skills within the group (e.g. focus on ‘I’ statements, bottom lining/laser speech, and disallowing advice-giving)

* In reflecting on your group Health and Wellness coaching experience as reported earlier in this survey, how IMPORTANT was this competency?

The response options for importance are:

- ◯ Not Important
- ◯ Somewhat Important
- ◯ Important
- ◯ Very Important

* In reflecting on your group Health and Wellness coaching experience as reported earlier in this survey, how FREQUENTLY was this competency performed?

The response options for frequency are:

- ◯ Never
- ◯ Infrequently
- ◯ Occasionally
- ◯ Frequently
- ◯ Very frequently

2.2.2.2 Elevate the group’s shared wisdom by modeling coaching techniques (e.g. open-ended questions, reflections, affirmations, and intentional silence)

* In reflecting on your group Health and Wellness coaching experience as reported earlier in this survey, how IMPORTANT was this competency?

The response options for importance are:

- ◯ Not Important
- ◯ Somewhat Important
- ◯ Important
- ◯ Very Important

* In reflecting on your group Health and Wellness coaching experience as reported earlier in this survey, how FREQUENTLY was this competency performed?

The response options for frequency are:

- ◯ Never
- ◯ Infrequently
- ◯ Occasionally
- ◯ Frequently
- ◯ Very frequently

Group Health & Wellness Coaching Validation Survey

2.2.3 Provide context and manage participant expectations

2.2.3.1 Summarize topics and segue to next topic intentionally

In reflecting on your group Health and Wellness coaching experience as reported earlier in this survey, how IMPORTANT was this competency?

The response options for importance are:

- ◯ Not Important
- ◯ Somewhat Important
- ◯ Important
- ◯ Very Important

In reflecting on your group Health and Wellness coaching experience as reported earlier in this survey, how FREQUENTLY was this competency performed?

The response options for frequency are:

- ◯ Never
- ◯ Infrequently
- ◯ Occasionally
- ◯ Frequently
- ◯ Very frequently

2.2.3.2 Provide instructions for activities, eliciting participant understanding, and clarifying as needed

In reflecting on your group Health and Wellness coaching experience as reported earlier in this survey, how IMPORTANT was this competency?

The response options for importance are:

- ◯ Not Important
- ◯ Somewhat Important
- ◯ Important
- ◯ Very Important

In reflecting on your group Health and Wellness coaching experience as reported earlier in this survey, how FREQUENTLY was this competency performed?

The response options for frequency are:

- ◯ Never
- ◯ Infrequently
- ◯ Occasionally
- ◯ Frequently
- ◯ Very frequently

Group Health & Wellness Coaching Validation Survey

2.2.4 Encourage participation and group cohesion

2.2.4.1 Identify common themes and link participants into the topic being discussed

* In reflecting on your group Health and Wellness coaching experience as reported earlier in this survey, how IMPORTANT was this competency?

The response options for importance are:

- ◯ Not Important
- ◯ Somewhat Important
- ◯ Important
- ◯ Very Important

* In reflecting on your group Health and Wellness coaching experience as reported earlier in this survey, how FREQUENTLY was this competency performed?

The response options for frequency are:

- ◯ Never
- ◯ Infrequently
- ◯ Occasionally
- ◯ Frequently
- ◯ Very frequently

2.2.4.2 Harvest group wisdom and resources by identifying themes and new awarenesses

* In reflecting on your group Health and Wellness coaching experience as reported earlier in this survey, how IMPORTANT was this competency?

The response options for importance are:

- ◯ Not Important
- ◯ Somewhat Important
- ◯ Important
- ◯ Very Important

* In reflecting on your group Health and Wellness coaching experience as reported earlier in this survey, how FREQUENTLY was this competency performed?

The response options for frequency are:

- ◯ Never
- ◯ Infrequently
- ◯ Occasionally
- ◯ Frequently
- ◯ Very frequently

2.2.4.3 Balance coach guidance with participant and group autonomy

* In reflecting on your group Health and Wellness coaching experience as reported earlier in this survey, how IMPORTANT was this competency?

The response options for importance are:

- ◯ Not Important
- ◯ Somewhat Important
- ◯ Important
- ◯ Very Important

* In reflecting on your group Health and Wellness coaching experience as reported earlier in this survey, how FREQUENTLY was this competency performed?

The response options for frequency are:

- ◯ Never
- ◯ Infrequently
- ◯ Occasionally
- ◯ Frequently
- ◯ Very frequently

2.2.4.4 Incorporate a range of appropriate group activities, such as open group discussion, round robin, brainstorming, and breakout groups

* In reflecting on your group Health and Wellness coaching experience as reported earlier in this survey, how IMPORTANT was this competency?

The response options for importance are:

- ◯ Not Important
- ◯ Somewhat Important
- ◯ Important
- ◯ Very Important

* In reflecting on your group Health and Wellness coaching experience as reported earlier in this survey, how FREQUENTLY was this competency performed?

The response options for frequency are:

- ◯ Never
- ◯ Infrequently
- ◯ Occasionally
- ◯ Frequently
- ◯ Very frequently

2.2.4.5 Facilitate a sense of belonging and cultivate interdependence among all group participants

* In reflecting on your group Health and Wellness coaching experience as reported earlier in this survey, how IMPORTANT was this competency?

The response options for importance are:

- ◯ Not Important
- ◯ Somewhat Important
- ◯ Important
- ◯ Very Important

* In reflecting on your group Health and Wellness coaching experience as reported earlier in this survey, how FREQUENTLY was this competency performed?

The response options for frequency are:

- ◯ Never
- ◯ Infrequently
- ◯ Occasionally
- ◯ Frequently
- ◯ Very frequently

2.2.4.6 Ensure adequate and appropriate attention for each group participant

* In reflecting on your group Health and Wellness coaching experience as reported earlier in this survey, how IMPORTANT was this competency?

The response options for importance are:

◯ Not Important

◯ Somewhat Important

◯ Important

◯ Very Important

* In reflecting on your group Health and Wellness coaching experience as reported earlier in this survey, how FREQUENTLY was this competency performed?

The response options for frequency are:

◯ Never

◯ Infrequently

◯ Occasionally

◯ Frequently

◯ Very frequently

2.2.4.7 Recognize and create experiences that facilitate discovery while accommodating different learning styles and preferences

* In reflecting on your group Health and Wellness coaching experience as reported earlier in this survey, how IMPORTANT was this competency?

The response options for importance are:

◯ Not Important

◯ Somewhat Important

◯ Important

◯ Very Important

* In reflecting on your group Health and Wellness coaching experience as reported earlier in this survey, how FREQUENTLY was this competency performed?

The response options for frequency are:

◯ Never

◯ Infrequently

◯ Occasionally

◯ Frequently

◯ Very frequently

2.2.4.8 Promote collaborative discussions by encouraging the acknowledgment of other's contributions

* In reflecting on your group Health and Wellness coaching experience as reported earlier in this survey, how IMPORTANT was this competency?

The response options for importance are:

◯ Not Important

◯ Somewhat Important

◯ Important

◯ Very Important

* In reflecting on your group Health and Wellness coaching experience as reported earlier in this survey, how FREQUENTLY was this competency performed?

The response options for frequency are:

◯ Never

◯ Infrequently

◯ Occasionally

◯ Frequently

◯ Very frequently

Group Health & Wellness Coaching Validation Survey

2.2.5 Group development and evolution

2.2.5.1 Understand and facilitate a participant's evolution from a self-focus to a collective group focus in regard to goal achievement

* In reflecting on your group Health and Wellness coaching experience as reported earlier in this survey, how IMPORTANT was this competency?

The response options for importance are:

- ◯ Not Important
- ◯ Somewhat Important
- ◯ Important
- ◯ Very Important

* In reflecting on your group Health and Wellness coaching experience as reported earlier in this survey, how FREQUENTLY was this competency performed?

The response options for frequency are:

- ◯ Never
- ◯ Infrequently
- ◯ Occasionally
- ◯ Frequently
- ◯ Very frequently

2.2.5.2 Recognize that bonding and investment of members builds engagement through the practice of altruism

* In reflecting on your group Health and Wellness coaching experience as reported earlier in this survey, how IMPORTANT was this competency?

The response options for importance are:

- Not Important
- Somewhat Important
- Important
- Very Important

* In reflecting on your group Health and Wellness coaching experience as reported earlier in this survey, how FREQUENTLY was this competency performed?

The response options for frequency are:

- Never
- Infrequently
- Occasionally
- Frequently
- Very frequently

2.2.5.3 Champion the group by regularly and consistently giving supportive feedback that is specific and timely

* In reflecting on your group Health and Wellness coaching experience as reported earlier in this survey, how IMPORTANT was this competency?

The response options for importance are:

- Not Important
- Somewhat Important
- Important
- Very Important

* In reflecting on your group Health and Wellness coaching experience as reported earlier in this survey, how FREQUENTLY was this competency performed?

The response options for frequency are:

- ◯ Never
- ◯ Infrequently
- ◯ Occasionally
- ◯ Frequently
- ◯ Very frequently

2.2.5.4 Understand and facilitate the phases of the group development process

* In reflecting on your group Health and Wellness coaching experience as reported earlier in this survey, how IMPORTANT was this competency?

The response options for importance are:

- ◯ Not Important
- ◯ Somewhat Important
- ◯ Important
- ◯ Very Important

* In reflecting on your group Health and Wellness coaching experience as reported earlier in this survey, how FREQUENTLY was this competency performed?

The response options for frequency are:

- ◯ Never
- ◯ Infrequently
- ◯ Occasionally
- ◯ Frequently
- ◯ Very frequently

Group Health & Wellness Coaching Validation Survey

2.2.6 Manage energy, emotions, and flow of the session

2.2.6.1 Attend to shifts in both individual and group energy (e.g. nonverbal communication signals)

* In reflecting on your group Health and Wellness coaching experience as reported earlier in this survey, how IMPORTANT was this competency?

The response options for importance are:

◯ Not Important

◯ Somewhat Important

◯ Important

◯ Very Important

* In reflecting on your group Health and Wellness coaching experience as reported earlier in this survey, how FREQUENTLY was this competency performed?

The response options for frequency are:

◯ Never

◯ Infrequently

◯ Occasionally

◯ Frequently

◯ Very frequently

2.2.6.2 Apply nonverbal communication appropriately to the group

* In reflecting on your group Health and Wellness coaching experience as reported earlier in this survey, how IMPORTANT was this competency?

The response options for importance are:

- Not Important
- Somewhat Important
- Important
- Very Important

* In reflecting on your group Health and Wellness coaching experience as reported earlier in this survey, how FREQUENTLY was this competency performed?

The response options for frequency are:

- Never
- Infrequently
- Occasionally
- Frequently
- Very frequently

2.2.6.3 Demonstrate empathy and honor emotions, recognizing the importance of respecting both individual and group boundaries

* In reflecting on your group Health and Wellness coaching experience as reported earlier in this survey, how IMPORTANT was this competency?

The response options for importance are:

- Not Important
- Somewhat Important
- Important
- Very Important

* In reflecting on your group Health and Wellness coaching experience as reported earlier in this survey, how FREQUENTLY was this competency performed?

The response options for frequency are:

◯ Never

◯ Infrequently

◯ Occasionally

◯ Frequently

◯ Very frequently

2.2.6.4 Manage emotions to create a safe container for the group, by naming and reflecting the emotion

* In reflecting on your group Health and Wellness coaching experience as reported earlier in this survey, how IMPORTANT was this competency?

The response options for importance are:

◯ Not Important

◯ Somewhat Important

◯ Important

◯ Very Important

* In reflecting on your group Health and Wellness coaching experience as reported earlier in this survey, how FREQUENTLY was this competency performed?

The response options for frequency are:

◯ Never

◯ Infrequently

◯ Occasionally

◯ Frequently

◯ Very frequently

2.2.6.5 Celebrate the forward progress of some, while remaining sensitive to others who may be 'stuck'

* In reflecting on your group Health and Wellness coaching experience as reported earlier in this survey, how IMPORTANT was this competency?

The response options for importance are:

- Not Important
- Somewhat Important
- Important
- Very Important

* In reflecting on your group Health and Wellness coaching experience as reported earlier in this survey, how FREQUENTLY was this competency performed?

The response options for frequency are:

- Never
- Infrequently
- Occasionally
- Frequently
- Very frequently

2.2.6.6 Use humor to raise or lighten group energy when it best serves the group process

* In reflecting on your group Health and Wellness coaching experience as reported earlier in this survey, how IMPORTANT was this competency?

The response options for importance are:

- Not Important
- Somewhat Important
- Important
- Very Important

* In reflecting on your group Health and Wellness coaching experience as reported earlier in this survey, how FREQUENTLY was this competency performed?

The response options for frequency are:

- ◯ Never
- ◯ Infrequently
- ◯ Occasionally
- ◯ Frequently
- ◯ Very frequently

2.2.6.7 Foster group and individual self-compassion

* In reflecting on your group Health and Wellness coaching experience as reported earlier in this survey, how IMPORTANT was this competency?

The response options for importance are:

- ◯ Not Important
- ◯ Somewhat Important
- ◯ Important
- ◯ Very Important

* In reflecting on your group Health and Wellness coaching experience as reported earlier in this survey, how FREQUENTLY was this competency performed?

The response options for frequency are:

- ◯ Never
- ◯ Infrequently
- ◯ Occasionally
- ◯ Frequently
- ◯ Very frequently

Group Health & Wellness Coaching Validation Survey

2.2.7 Manage challenging group participant behaviors (i.e. under or over-talking, interrupting, fixing, arguing, using inappropriate language, being culturally insensitive, veering off topic, etc.) escalating as necessary from general to specific, for example:

- **Reinforce group honoring of ground rules**
- **Redirect and reframe communication**
- **Openly name and address discord appropriately within the group as it occurs**
- **Have a private discussion with participant(s) outside of group**
- **Attend to patterns of group conflict and resistance**
- **Model skills for conflict management and navigation of difficult conversations**

* In reflecting on your group Health and Wellness coaching experience as reported earlier in this survey, how IMPORTANT was this competency?

The response options for importance are:

◯ Not Important

◯ Somewhat Important

◯ Important

◯ Very Important

* In reflecting on your group Health and Wellness coaching experience as reported earlier in this survey, how FREQUENTLY was this competency performed?

The response options for frequency are:

◯ Never

◯ Infrequently

◯ Occasionally

◯ Frequently

◯ Very frequently

Group Health & Wellness Coaching Validation Survey

2.3 Model active, mindful listening, and nonjudgmental presence

Overview: In addition to listening to verbal information shared by the group , the coach(es) is attuned to nonverbal cues, both from and between participants: expression, tone, emotions, and energy. The coach also notices relevant behaviors (or lack thereof). The coach uses mindful awareness to notice with curiosity and non judgment what is happening within the group , as well as what is happening within him/herself during coaching. Coach self-management is required when the coach finds him/herself “knowing” what the group needs. Finally, active listening involves using silence appropriately to “hold the space” and allowing group members time to reflect, process, and identify what emerges.

2.3.1 Take into account differing processing styles and pace

* In reflecting on your group Health and Wellness coaching experience as reported earlier in this survey, how IMPORTANT was this competency?

The response options for importance are:

◯ Not Important

◯ Somewhat Important

◯ Important

◯ Very Important

* In reflecting on your group Health and Wellness coaching experience as reported earlier in this survey, how FREQUENTLY was this competency performed?

The response options for frequency are:

◯ Never

◯ Infrequently

◯ Occasionally

◯ Frequently

◯ Very frequently

2.3.2 Demonstrate cultural sensitivity and accommodate different world views

* In reflecting on your group Health and Wellness coaching experience as reported earlier in this survey, how IMPORTANT was this competency?

The response options for importance are:

◯ Not Important

◯ Somewhat Important

◯ Important

◯ Very Important

* In reflecting on your group Health and Wellness coaching experience as reported earlier in this survey, how FREQUENTLY was this competency performed?

The response options for frequency are:

◯ Never

◯ Infrequently

◯ Occasionally

◯ Frequently

◯ Very frequently

NBHWC

Group Health & Wellness Coaching Validation Survey

2.4 Set goals, implement action commitments, and review progress

Overview: Through understanding participants' change readiness, recognizing and leveraging strengths, and supporting group members in designing appropriate action steps to overcome limitations, coaches move each group member toward gradual success with self-determined goals and long-term engagement. Tracking progress over time is strongly linked to
sustainable success, so group members learn to track their own behavior, problem-solve, and observe the impact of their actions. When reviewing such progress, the coach does not focus on the outcome, but rather emphasizes the group member's effort and discoveries during both
successes and setbacks. Coaches encourage participant's recognition of their own strengths and limitations, and reinforces the group members in supporting each other in this process.

2.4.1 Consistently integrate goal setting and progress reviews into sessions

* In reflecting on your group Health and Wellness coaching experience as reported earlier in this survey, how IMPORTANT was this competency?

The response options for importance are:

◯ Not Important

◯ Somewhat Important

◯ Important

◯ Very Important

* In reflecting on your group Health and Wellness coaching experience as reported earlier in this survey, how FREQUENTLY was this competency performed?

The response options for frequency are:

◯ Never

◯ Infrequently

◯ Occasionally

◯ Frequently

◯ Very frequently

2.4.2 Support accountability among group participants

* In reflecting on your group Health and Wellness coaching experience as reported earlier in this survey, how IMPORTANT was this competency?

The response options for importance are:

◯ Not Important

◯ Somewhat Important

◯ Important

◯ Very Important

* In reflecting on your group Health and Wellness coaching experience as reported earlier in this survey, how FREQUENTLY was this competency performed?

The response options for frequency are:

- Never
- Infrequently
- Occasionally
- Frequently
- Very frequently

2.4.3 Apply learning to real life goals and action, which may include debriefing of activities

* In reflecting on your group Health and Wellness coaching experience as reported earlier in this survey, how IMPORTANT was this competency?

The response options for importance are:

- Not Important
- Somewhat Important
- Important
- Very Important

* In reflecting on your group Health and Wellness coaching experience as reported earlier in this survey, how FREQUENTLY was this competency performed?

The response options for frequency are:

- Never
- Infrequently
- Occasionally
- Frequently
- Very frequently

2.4.4 Invite group members to normalize and reframe setbacks, obstacles, and challenges

* In reflecting on your group Health and Wellness coaching experience as reported earlier in this survey, how IMPORTANT was this competency?

The response options for importance are:

- ◯ Not Important
- ◯ Somewhat Important
- ◯ Important
- ◯ Very Important

* In reflecting on your group Health and Wellness coaching experience as reported earlier in this survey, how FREQUENTLY was this competency performed?

The response options for frequency are:

- ◯ Never
- ◯ Infrequently
- ◯ Occasionally
- ◯ Frequently
- ◯ Very frequently

2.4.5 Honor individual preferences for self-monitoring

* In reflecting on your group Health and Wellness coaching experience as reported earlier in this survey, how IMPORTANT was this competency?

The response options for importance are:

- ◯ Not Important
- ◯ Somewhat Important
- ◯ Important
- ◯ Very Important

* In reflecting on your group Health and Wellness coaching experience as reported earlier in this survey, how FREQUENTLY was this competency performed?

The response options for frequency are:

- ◯ Never
- ◯ Infrequently
- ◯ Occasionally
- ◯ Frequently
- ◯ Very frequently

2.4.6 Elicit commitment for actions to be taken before the next session

* In reflecting on your group Health and Wellness coaching experience as reported earlier in this survey, how IMPORTANT was this competency?

The response options for importance are:

- ◯ Not Important
- ◯ Somewhat Important
- ◯ Important
- ◯ Very Important

* In reflecting on your group Health and Wellness coaching experience as reported earlier in this survey, how FREQUENTLY was this competency performed?

The response options for frequency are:

- ◯ Never
- ◯ Infrequently
- ◯ Occasionally
- ◯ Frequently
- ◯ Very frequently

Group Health & Wellness Coaching Validation Survey

2.5 Enhance social support

Overview: In addition to providing support during group coaching sessions, the coach(es) assists the group members in developing supportive relationships and identifying family and community resources. The coach helps the group to build a support system—relationships, tools, resources, environments—that enable ongoing success after the group coaching program ends.

2.5.1 Facilitate participants in developing and accessing social support inside and outside of the group

* In reflecting on your group Health and Wellness coaching experience as reported earlier in this survey, how IMPORTANT was this competency?

The response options for importance are:

◯ Not Important

◯ Somewhat Important

◯ Important

◯ Very Important

* In reflecting on your group Health and Wellness coaching experience as reported earlier in this survey, how FREQUENTLY was this competency performed?

The response options for frequency are:

◯ Never

◯ Infrequently

◯ Occasionally

◯ Frequently

◯ Very frequently

2.5.2 Bring awareness to outlets participants may utilize outside of sessions, such as social media, to share shifts, breakthroughs, or challenges they experience between sessions

* In reflecting on your group Health and Wellness coaching experience as reported earlier in this survey, how IMPORTANT was this competency?

The response options for importance are:

- ◯ Not Important
- ◯ Somewhat Important
- ◯ Important
- ◯ Very Important

* In reflecting on your group Health and Wellness coaching experience as reported earlier in this survey, how FREQUENTLY was this competency performed?

The response options for frequency are:

- ◯ Never
- ◯ Infrequently
- ◯ Occasionally
- ◯ Frequently
- ◯ Very frequently

2.5.3 Facilitate participants envisioning how to create needed support outside of the group, transferring the skills they've learned in the group

* In reflecting on your group Health and Wellness coaching experience as reported earlier in this survey, how IMPORTANT was this competency?

The response options for importance are:

◯ Not Important

◯ Somewhat Important

◯ Important

◯ Very Important

* In reflecting on your group Health and Wellness coaching experience as reported earlier in this survey, how FREQUENTLY was this competency performed?

The response options for frequency are:

◯ Never

◯ Infrequently

◯ Occasionally

◯ Frequently

◯ Very frequently

NBHWC

Group Health & Wellness Coaching Validation Survey

Are there any competencies not included in this survey that you think are important for group coaching?

Thank you again for your time and assistance with this validation survey!
